# Searching for Synergies: Matrix Algebraic Approaches for Efficient Pair Screening

**DOI:** 10.1371/journal.pone.0068598

**Published:** 2013-07-25

**Authors:** Philip Gerlee, Linnéa Schmidt, Naser Monsefi, Teresia Kling, Rebecka Jörnsten, Sven Nelander

**Affiliations:** 1 Sahlgrenska Cancer Center, University of Gothenburg, Gothenburg, Sweden; 2 Mathematical Sciences, University of Gothenburg and Chalmers University of Technology, Gothenburg, Sweden; 3 Department of Immunology, Genetics and Pathology and Science for Life Laboratory, Uppsala University, Uppsala, Sweden; Queen's University Belfast, United Kingdom

## Abstract

Functionally interacting perturbations, such as synergistic drugs pairs or synthetic lethal gene pairs, are of key interest in both pharmacology and functional genomics. However, to find such pairs by traditional screening methods is both time consuming and costly. We present a novel computational-experimental framework for efficient identification of synergistic target pairs, applicable for screening of systems with sizes on the order of current drug, small RNA or SGA (Synthetic Genetic Array) libraries (>1000 targets). This framework exploits the fact that the response of a drug pair in a given system, or a pair of genes' propensity to interact functionally, can be partly predicted by computational means from (i) a *small* set of experimentally determined target pairs, and (ii) pre-existing data (e.g. gene ontology, PPI) on the similarities between targets. Predictions are obtained by a novel matrix algebraic technique, based on cyclical projections onto convex sets. We demonstrate the efficiency of the proposed method using drug-drug interaction data from seven cancer cell lines and gene-gene interaction data from yeast SGA screens. Our protocol increases the rate of synergism discovery significantly over traditional screening, by up to 7-fold. Our method is easy to implement and could be applied to accelerate pair screening for both animal and microbial systems.

## Introduction

System-scale chemical and genetic screens have progressed from testing single targets to testing combinations of targets. Pairwise tests can reveal functional couplings, such as drug-drug synergism and pathway modules, that cannot be captured by single target screens. In a typical setting, the functional interaction between two targets 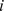 and 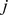 (drugs or genes) is calculated as an interaction score 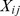, commonly defined as:
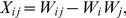
(1)where 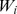 and 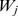 are the relative phenotypes after perturbations of single targets 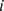, 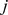 and 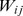 is the response to perturbation of the 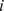 and 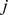 combination.

System-scale mapping of all interaction scores 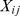 can serve several important purposes. First, positive and negative values of 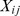 can be interpreted within the framework of epistasis analysis to deduce pathway relationships between the targets 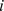 and 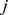, or to define functional modules in the system [1]–[7]. Second, both negative and positive interactions are of considerable therapeutic interest. Negative interactions reveal synergistic target pairs that can increase efficiency and widen the therapeutic window of a treatment. Positive interactions can reveal redundant target pairs that may slow down the acquisition of drug resistance [8], [9]. Screens in several cellular systems, e.g. cancer cells, have revealed that combination effects are prevalent [10]; thus, mapping interaction scores in cellular systems presents an important challenge for systems biology [11]–[14].

In a traditional pair screening process, an interaction score, 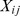, is experimentally obtained for *every* pair 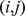, and pairs are considered interacting if the interaction score (or some relevant statistic that captures functional coupling) exceeds a threshold. Exhaustive screening is a very costly strategy, since the number of experiments needed grows quadratically with the number of targets, 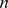. The largest pair screening reported [4] is of a magnitude of 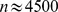. However, to screen drug libraries (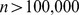) or human shRNA libraries (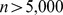), the experimental burden would be prohibitive for standard labs.

Here, we therefore recast the screening problem in terms of a different goal: can we find a *reasonably high fraction* of all synergistic pairs (e.g. 75%), by testing a *relatively low fraction* of all pairs (e.g. 20%)? The acceleration of pairwise interaction mapping was previously proposed in the context of pulldown experiments for PPI mapping [15], [16], but also methods specific to genetic interactions have been proposed [17], [18]. Our method differs from these in that it exploits properties of interaction networks common to both PPIs and genetic networks, and hence has wider applicability. In addition, the method does not assume a particular experimental design as in pulldown experiments.

We introduce a mathematical notion of screening efficiency and methods to maximize this efficiency, based on alternation between gradual experimental testing and a matrix algebraic technique to predict synergism. The functioning of this novel algorithm does not rely on the degree of target specificity, or a particular choice of interactions measure, and using several data sets from yeast and cancer cell lines, we demonstrate that our method greatly improves screening efficiency and is both computationally efficient and easy to implement. Further, the performance of the algorithm can be improved by including similarity between drugs/genes, such as target of action or functional interactions.

## Results

### Quantifying screening efficiency by the fractional discovery rate

To characterize screening efficiency, we propose to use the *fractional discovery rate*. Since the algorithms we propose are stochastic in nature we suggest to use a metric which quantifies the average behaviour of an algorithm when applied to a certain data set.

**Figure 1:**
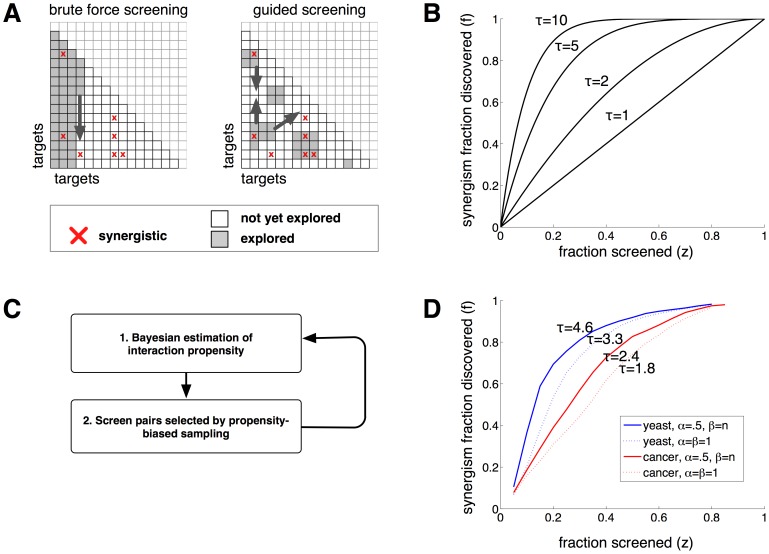
Efficient experimental screening. A: Principal difference between systematic screening (testing all pairs sequentially) and guided screening (letting discovered synergistic pairs, marked as red X's guide the subsequent steps of the screening process). B: We characterize the screening process by the fractional discovery rate 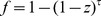 attained at experimental fraction 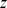, where 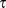 denotes the screening efficiency. In a screening process with high 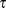 value, a large fraction of all synergies is found by testing a small fraction of all possible target pairs. 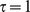 corresponds to systematic screening. C: A simple protocol to increase 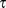 is to direct the screen towards targets that seem prone to synergism. For this, we propose a sampling protocol based on Bayesian estimates of a target's propensity to interact. D: We use a beta-prior with parameters 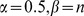, where 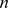 is the number of targets. A flat prior (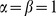) reduces performance.

Consider the following hypothetical scenario: an idealized screen is carried out in which the experimenter tests a growing fraction 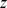 of all possible pairs of drugs/genes. At a given 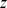 a fraction 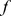 of all synergistic pairs have been discovered. Now imagine that we repeat the screening process many times and calculate the average fraction of discovered pairs at a given 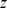, described by the curve 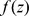 (Figure 1A,B). We define the fractional discovery rate as the derivative 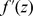. If an experimenter screens in a systematic, “brute force” fashion, the expected value of the fractional discovery rate will be given by 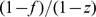, i.e. the ratio between the remaining fraction of synergies 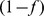 and the remaining screenable fraction 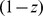. This implies that the relation
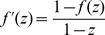
(2)holds for all 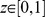.

Let us now consider a screening principle, such as our proposed method, that enriches for synergism. We summarize this enrichment by a factor of 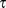, where now instead
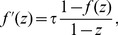
(3)i.e. the fractional discovery rate is 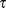 times higher as compared to “brute force” experimentation. We refer to 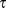 as the *screening efficiency*. We solve this differential equation, with boundary conditions 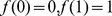, and obtain the explicit relationship:
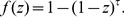
(4)For 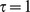 this function simply describes a line with slope 1, going from (0,0) to (1,1). Efficient screening procedures should identify a large fraction of synergies from a relative low fraction of all pair experiments, thus resulting in higher values of 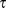 (Figure 1B). An oracle screen (knowledge of which pairs are synergistic) achieves the maximum possible 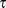, given by 1 divided by the prevalence of synergistic pairs.

We will now discuss how to construct screening procedures that improve synergism discovery. We thus proceed to formulate an experimental protocol that incorporates the following three ideas; (i) concurrent estimation of the synergism propensity; (ii) a novel interaction score imputation framework which performs well in cases where the screened fraction is low and can take biological database information into account, and; (iii) an adaptive strategy that toggles between principles (i) and (ii) to optimize screening efficiency. These three components of the experimental protocol are described in the sections below.

We assess the performance on nine data sets, comprising seven cancer cell lines and two yeast data sets (Methods and Table 1) We reason that achieving a high value of 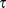 across a range of screens of different size and type of data should extrapolate to future screens.

**Table 1:** Benchmarking data sets.

Data set	Assay	Number of targets
Costanzo et al.	Synthetic Genetic Array	4457 (944 with prior information used here)
Schuldiner et al.	Synthetic Genetic Array - like method	427
Colon cancer cell line (HCT116)	Drug-drug interaction	190
Lung cancer cell line (A549)	Drug-drug interaction	190
Glioblastoma cell lines (T98G, U343MG, U87MG, U373MG, A172)	Drug-drug interaction	31 for each cell line

### Propensity-based sampling improves screening efficiency

Based on the assumption that some targets are more likely to interact than others (so-called “hubs” in a system), one should be able to increase the screening efficiency, 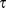, by prioritizing targets that have been identified as synergistic in the early phases of the screen. In previous work, Myers and co-workers have used such methods to predict the number of interactors of yeast genes [4].

Here, we formulate an concurrent estimation scheme to prioritize targets likely to be involved in synergies. We denote a target 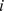
's propensity to interact by 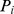, 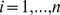. Given current estimates of the 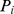, we select pairs 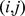 for testing with probability 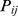, where
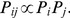
(5)This simple screening protocol is random, but biased towards the likely hubs of interactions (Algorithm 1, Methods). To make the screening protocol adaptive, we use a Bayesian estimate of 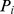, as follows. We first assume that the likelihood to observe 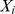 synergies for target 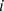, is binomial distributed with parameters 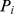, 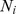. We subsequently assume that the parameter of this distribution, 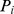, was drawn from a conjugate prior beta distribution with parameters 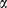 and 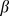. The estimate of 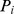 is thus given by:
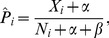
(6)where 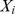 is the number of synergies found, and 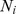 the total number of interactions tested for gene (or drug) 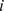. This estimate is the mean of the posterior distribution of 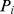, given a beta-distributed prior for 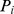 with parameters 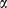 and 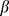. Maximum marginal likelihood estimates of 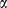 and 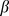 from data suggest using 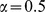 and 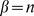 in our protocol (see Methods). This corresponds to a prior belief that relatively few targets constitute hubs of interaction. Applying this simple protocol to our set of 9 different interaction score matrices, and averaging over a large number of realisations, we achieve a 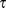 in the range 2.0 to 4.6 (Figure 1D). At experimental fraction 20%, we are able to discover 37% to 67% of all synergies (compared with 20% for brute-force screening). Using of a flat prior (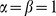, prior belief that roughly half the targets interact) reduces 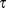 substantially (Figure 1D). The difference in performance on the different data sets is mainly attributed to the size of the data sets, where the method performs better on larger sets of data (see Discussion).

### Matrix imputation for highly incomplete interaction score data

To improve screening efficiency further, we use matrix completion to predict likely synergies from limited amounts of screening data. Matrix completion methods to impute missing values have been tested on interaction score matrices [19], [20] when a small percentage of data is missing. Recent results, however, have shown that surprisingly few entries are needed for imputation in cases where a matrix possesses some underlying ‘block-like’ structure [21], [22]. This type of structure is know to be prevalent among interaction score matrices [2], [23].

#### Prediction of interaction scores using set projections

We proceed to define a customized matrix completion method for interaction score data which, unlike standard matrix completion algorithms, encompasses an option to include prior information on functional similarity between targets. For instance, our method can be used to include information on shared mechanism of action between drugs, or shared pathway membership between genes, both likely to give rise to similar interaction behavior. We give a brief description of the method here (details in Methods). The interaction score matrix 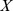 represents a point in the space of all symmetric 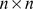 matrices. We assume that this point lies in the intersection of three convex sets, termed 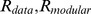 and 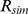, each encoding a different type of evidence:

1. The set 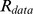 contains all symmetric matrices that agrees with the currently available data, within an error tolerance level (Methods, eq. 8). For example, if half the entries of the matrix are known, then 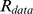 consists of all matrices where the known entries (within error) are equal to the data, and where all other entries are allowed to vary freely.
2. The set 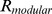 contains all matrices with a sufficiently block-like structure. Both gene-gene and drug-drug interaction scores form clusters (blocks), thought to reflect some degree of intrinsic modular organization of cellular pathways [24]. Following [21], we define modularity as a constraint on the nuclear norm of the matrix 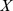 (Methods, eq. 9).
3. The set 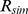 contains all matrices 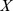 that conform with externally defined information on functional similarities between the targets. We expect some degree of correlation between the rows/columns 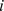 and 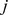 in 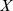, when targets 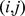 have a similar biological function [4]. We represent functional similarity by a matrix 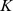, with the property 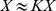, derived from data sources such as PPI networks, GO terms and drug mechanism of action (Methods, eq. 10).

**Figure 2:**
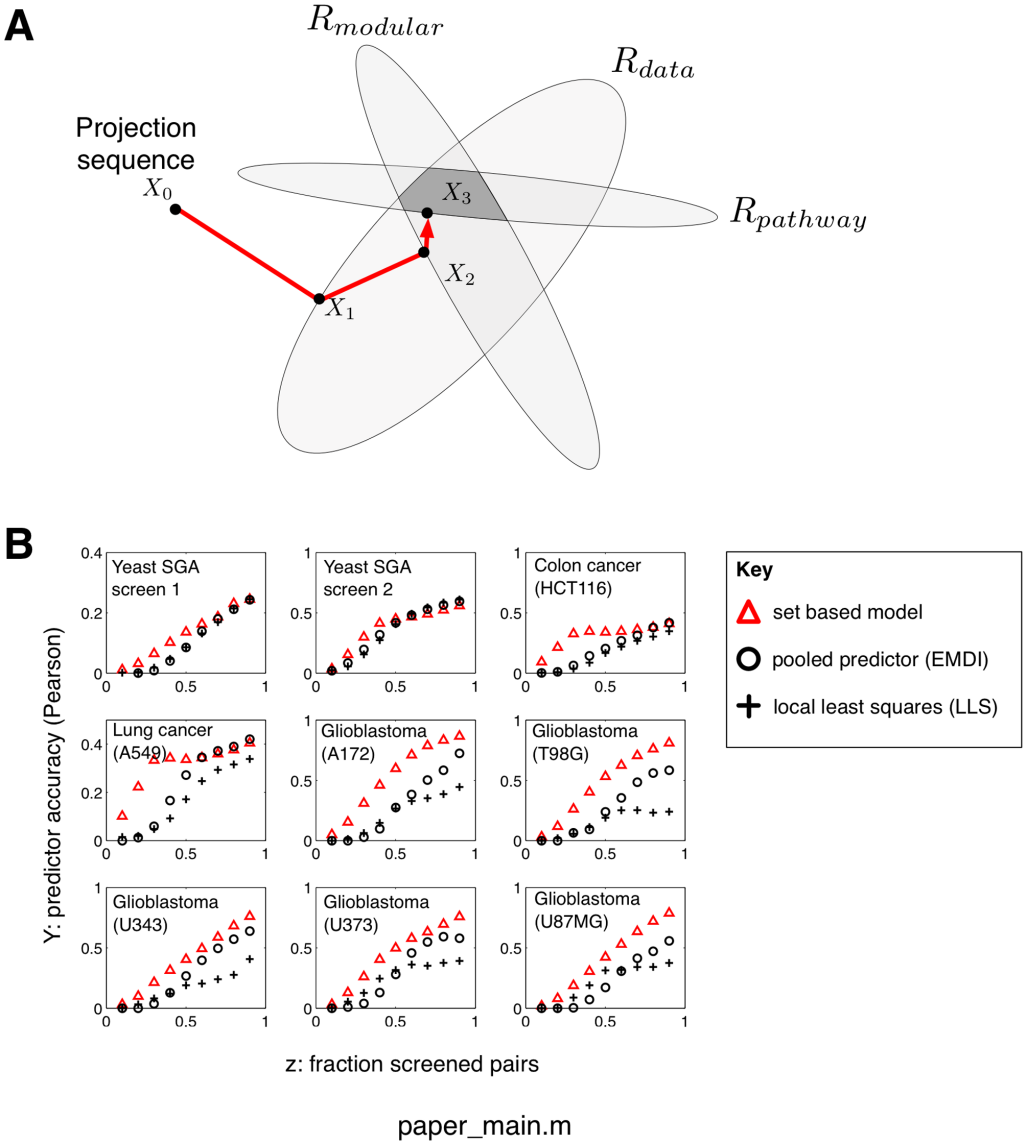
Predicting synergism scores from highly incomplete data via cyclical set projection. A: To improve screening efficiency further, we introduce a projection-based predictor of synergism scores. An initial guess of a synergism score matrix 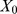 is projected first onto the set 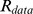, which corresponds to known interaction scores, then onto the set 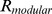, which contains matrices of approximately low rank, and finally onto 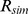, holding the matrices consistent with known functional similarity. The projections are applied cyclically until convergence to a final prediction of 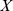 is reached, which is guaranteed due to convexity of the three sets (here illustrating convergence in one iteration). B: Prediction accuracy in five glioblastoma cell lines and reference data sets. Comparison between our projection-based method and two state-of-the-art methods for interaction score imputation methods, LLS and EMDI. Generally, set based projections outperform the other methods (predictions correlate more with true values), especially when the screened fraction 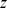 is small.

We find a feasible point, i.e. a solution 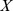 located in the intersection 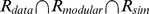, by a cyclical sequence of projections onto these convex sets from a given starting point (Figure 2A). This iterative algorithm is highly efficient and can be applied to data where the number of targets 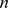 is quite large, ranging up to a couple of thousand targets (see Implementation in Methods).

#### Comparison to existing matrix completion methods

To assess the performance of the novel imputation technique, we compare our method to two other techniques: (i) The recently proposed Local Least Squares (LLS) [19] which was advantageously compared to other approaches such as Bayesian Principal Components Analysis [25]; (ii) A meta-predictor, EMDI, which combines LLS and several other methods by a weighted average [20]. For each method, we separately assess the prediction accuracy for each sample fraction 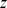 of observed matrix entries, 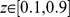 (randomly sampled from the data). Our model gives more accurate predictions from sparse data (10–20% observation) across all data sets (Figure 2B), and is highly competitive even for larger sample fractions 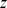. We compared the prediction accuracy measured as Pearson correlation over 20 independent runs. This gave a strong significance (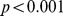) in 7 datasets (both yeast SGA screens, colon cancer, lung cancer, and A172, T98G, U343 glioma cells), and a moderate/weak significance in 2 datasets (U373 and U87; 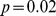 and 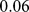 respectively) For our comparisons, we also considered the APN method by Battle et al. [5]. However, this method is specifically aimed at predicting buffering/antagonistic relationships, and is computationally heavy.

### Combining propensity-based sampling with prediction-driven screening

**Figure 3:**
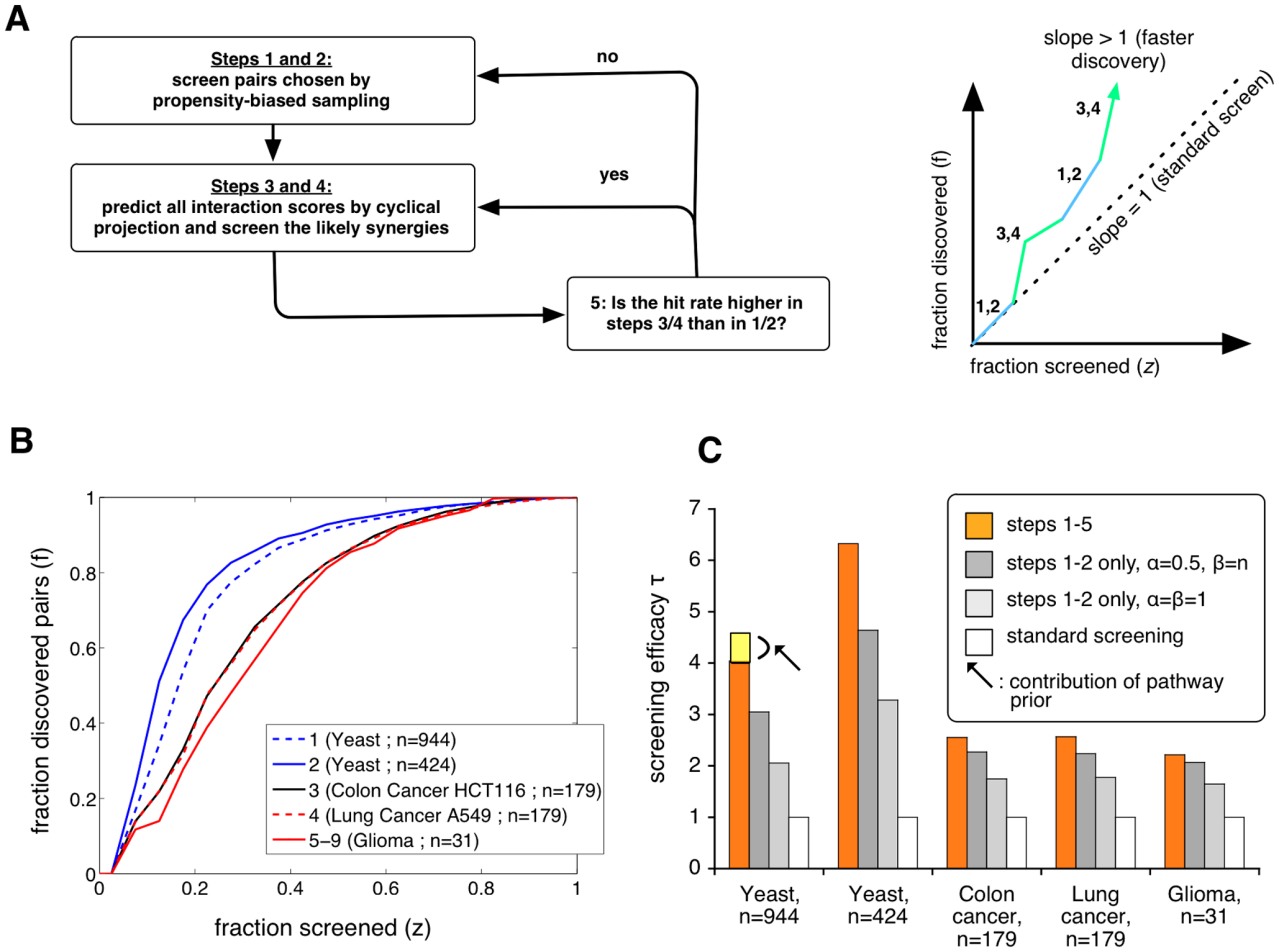
Improving screening efficiency by combining propensity-based sampling with interaction score prediction via matrix completion. A: We extend the simpler protocol (propensity-based sampling only, Figure 1C), adding a projection-based predictor to choose likely synergistic pairs (steps 3 and 4). If the prediction-driven screening discovery rate is higher than the preceding propensity-based screening, a new prediction-driven screening cycle is started (step 5). We switch between propensity-based sampling and prediction to increase the fractional discovery rate. B: Fractional discovery rate across 9 data sets show marked improvement over brute-force screening. C: Estimates of the screening efficiency 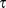 demonstrate that the full protocol (steps 1–5) gives better performance than propensity-based sampling only (steps 1–2). Yellow block: additional contribution by projecting onto 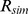 in the largest yeast SGA screen.

Our final screening procedure (Algorithm 2, Methods) combines and alternates between the two different “search modes” described above: (i) propensity-based random sampling, biased toward untested pairs comprising targets that have hitherto exhibited a high propensity for interacting with other targets (in steps 1 and 2); and (ii) greedy collection of target pairs for which matrix completion has predicted a high degree of synergism (steps 3 and 4). The balance between the two search modes is determined by their performance (step 5) (Figure 3A).

We find that combining interaction prediction and propensity-based sampling to guide the screening process results in a screening efficiency 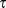 much better than the propensity-based sampling alone (Figure 3C). For instance, our procedure detects 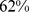 and 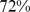 of the synergistic pairs in the yeast data sets by testing only 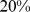 of the interactions (Figure 3B). This drops to 46% and 67% when we use propensity-based sampling only (compared with expected 20% for brute-force screening). For the largest data set [4], we see a 4.5-fold increase in efficiency 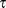 for the full protocol over brute-force screening, which drops to about four-fold when the prediction method uses no prior information, and to only three-fold with propensity-based sampling alone (Figure 3D).

## Discussion

### Performance and data requirements: the impact of modularity and screen size

We note that screening efficiency is most improved for the larger screens. Intuitively, for small systems, there is not much room for targeted screening to improve over brute-force methods. However, as the number of possible interaction pairs grows, it becomes more important to choose experiments carefully to speed up detection. More formally, imputation methods based on nuclear norm constraints (as in our 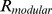) work best when the matrix rank 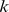 is much lower than the matrix size 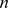. A theoretical result by Candes et al. [26] states that sample size requirements to make accurate predictions is proportional to 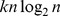. Assuming the number of modules (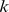) remains relatively constant for the different biological systems, 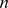 is thus the major determining factor for screening efficiency. We repeat the analysis on subsets of the largest data set, showing that screening efficiency indeed is proportional to the number of targets (Figure S1 in File S1). Thus, we expect the screening efficiency obtained with our protocol will grow with the number of targets.

One reason why our prediction approach performs well plausibly lies in the fact that all data sets we analyze exhibit a strong degree of modularity. Previously, both the two yeast data sets and the HCT116 and A549 cancer cell lines have been shown to contain functional clusters [4], [13]. To assess whether our own measurements in five glioma cell lines also show some functional modularity, we calculated the correlation (in the 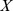 matrix) between drugs that belonged to the same vs. different categories. This analysis confirmed that drugs in the same category (e.g. RTK inhibitors) have higher correlations (Figure S2 in File S1).

In terms of other approaches proposed for accelerated pair screening our method bears some resemblance to the algorithm suggested by Lappe et al. [15]. That method was designed for exploring PPI networks, and gathers information during the screen to accelerate the process. The method exploits the fact that the network contains hubs with high connectivity, and uses as bait for the next experiment the protein that has been seen as prey most often so far. That is similar to our propensity based approach, but only works in the case where a target is tested against all other targets, as is the case of mass spectrometry pull-down experiments. Our method is different, and in a sense more refined, since it not only selects an entire row of the matrix, but a specific matrix element for testing.

The other component of our algorithm, which allows for the incorporation of prior knowledge about the targets, is somewhat similar to the method proposed by Wong et al. [17]. That method uses a wide variety of prior data such as subcellular localisation of the proteins, chromosomal distance etc. With this type of information they can predict the probability that a gene pair exhibits synthetic sick or lethal interactions. However their method is not suited for a screening process and is not optimised for handling incremental data. The main conclusions when comparing our algorithm with other methods is hence that it combines both a subroutine for defining single experiments based on previous findings in the screen, and allows for the incorporation of prior knowledge about the targets.

In a natural sense every discovery algorithm is limited by the type of assumptions that are made during the construction of the method, or in other words what type of patterns the data is expected to contain. In our case we have exploited the fact that interactions matrices tend to exhibit block-like structure, which mathematically corresponds to a low matrix rank, and also made use of the observation that certain hubs exists in the data. This implies that interactions which deviate from these patterns will be less likely to be detected, and this means that the algorithm is not geared towards “true” discovery, but limited by the assumptions made.

Another difficulty that arises when screening a novel system is to decide on an appropriate synergy threshold value, which effectively determines the number of targets in the screen. Data sets from many diverse system do however suggest that interaction scores have a charactersitic long-tailed, non-normal distribution, which lends some hope to transferring knowledge from one system to another. In most cases some preliminary data is also available, e.g. from the SGA data in yeast [4] we could derive a reasonable threshold value (see Methods), and this information can be carried over to other genetic systems.

## Conclusions

We have presented a novel method for screening of gene or drug-pairs with the aim of finding synergistic interactions as quickly and cost-efficiently as possible. We expect that advanced matrix imputation methods and prediction based screening procedures, as outlined here, may find several applications. For the five cancer cell lines here analyzed, the proposed methods can serve to rapidly map the interaction landscape for multiple drugs, helping guide discovery screens, and defining combination therapies that overcome some of the shortcomings of current monotherapies for cancer.

We conclude that our approach exhibits good performance on real experimental data. The proposed approach is distinct from previous matrix completion methods, since it also incorporates prior molecular information, and also distinct from methods that rely completely on molecular data [27]–[30]. In principle, the approach can be generalized to incorporate additional constraining sets to further improve the solution; this is reserved for future work.

The developed method is geared toward rapid discovery of synergistic pairs, and, in order to achieve this, modular and structural similarities between targets are exploited. The method is thus more likely to discover synergistic interactions that follow this modular pattern, whereas “unexpected” interactions will be harder to find.

Future directions include the exploration of higher order combinations [14], and to introduce improved, target specific estimation of the propensities 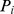, by for example taking into account the observed negative correlation between single mutant fitness and number of interactions [4]. It may also be interesting to investigate formal techniques for experimental planning [31], [32] and refine strategies to define our functional similarity matrix, 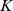 by including e.g. drug side-effect similarity [33]. These measures might improve screening efficiency even further.

## Methods

### Preparation and generation of benchmarking data sets

#### Gold standard/public data

Our data consists of 4 publicly available data sets (Table 1 and main text). The first two sets of measurements that we study are standard two-gene synthetic gene array data [4], [19], which both contain interaction scores (eq. 1) defined in terms of yeast viability under gene single/double gene knockouts. We used interaction scores as provided, without further normalization, obtained from the supplements of Costanzo et al. [4] (SGA experiments, using the rigorous cutoff preparation of the data; and SGA/ESP data as provided in the supplement of Colm et al. [19]). We used QQ plots against a normal distribution to choose a point where negative interaction scores deviated significantly from a normal distribution. This gave us a threshold value roughly 3 standard deviations from the mean, giving a prevalence of synergies of 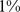. The next two data sets were obtained from Zalicus (previously CombinatoRx, a company that pursues drug pair screening) and represent drug pair responses in HCT116 and A549 colon cancer and lung cancer cells, respectively. Here, the interaction scores quantify drug-drug interaction across multiple doses, using a customized metric defined as in Lehar et al. [13]. For the CombinatoRx data, we used the synergism thresholds defined in the original publication (an S-index less than −0.29) which corresponds to a prevalence of synergies of 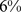.

#### Experiment in five glioblastoma cell lines

In addition, we generated data for five glioblastoma cell lines, as follows. Glioma cell line T98G was obtained from ATCC and A172, U-343MG, U-373MG and U-87MG were obtained from Cell lines Services, Germany. All cell lines were grown in monolayer and maintained in high-glucose (4.5 g/l) DMEM supplemented with 10% FBS (Fetal Bovine Serum), 1% PEST (Penicillin/Streptomycin) and 2 mM L-Glutamine and incubated at 37°C with 5% CO_2_ in a New Brunswick Galaxy R Incubator. For the experiments we selected a set of 31 compounds, some of which were selected uniformly at random from a library, and some of which had a similar or related mechanism of action. The drugs used are listed in Supplementary Table 1 in File S1. Tumor cells were plated at 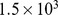 cells/well in a TPP 96-well plate 24 hours prior to treatment. Cells were treated with drugs diluted in media, single or in combination, and incubated for 48 hours. For combinations, 4 replicates were performed with 3–6 replicate negative controls of equal amount of DMSO (0.1–0.2%). Viability studies were performed using the alamar blue assay (Invitrogen Corp.). At end of experiment cells were incubated for 4 hours with alamar blue reagent (Invitrogen Corp.) for cell viabilty measurements. Fluorescence was read at Exc544/Em590 on a microplate reader (SPECTRAmax GEMINI XS, Molecular Devices). From viability assays, we quantified the drug response as the ratio 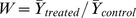, where 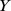 represents the fluorescence signal and bar represents average across replicates. We measured interaction scores for 465 drug pairs (corresponding to all pairs chosen from 31 compounds) using eq. (1), which is usually referred to as the Bliss interaction score. To identify synergies we proceed as with the public SGA screens, lowered the threshold to 1.5 standard deviations, and obtain 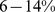 synergies in the different cell lines.

### Estimating a target's marginal propensity to interact

A target's propensity to interact is estimated concurrently during the screen using the formula
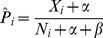
(7)where 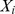 is the number of synergies found so far, 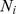 the total number of interactions tested for gene (or drug) 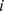, and we assume a beta-prior, with parameters 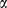 and 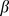, for a target's propensity to interact. The prior mean, 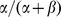, signifies the *a priori* expected interaction frequency of each target, here assumed to be the same for all targets.

We estimated the parameters 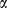 and 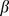 from the data sets using a maximum marginal likelihood estimate of the probability distribution 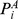, i.e. the probability to find a gene (or drug) with 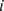 synergistic interactions in data set 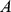 (Empirical Bayes) [34]. The obtained values were for 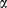 in the range 0.26–1.05 and 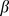 close to the number of targets (genes or drugs) 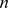. As a rule of thumb, we therefore suggest to use 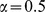 (the median observed value for all data sets) and 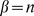 as a prior in our protocol. These values of 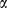 and 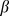 correspond to a prior skewed toward few interaction hubs.

We also compare the screening procedure obtained with a so-called flat prior (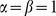). This prior corresponds to a prior belief that half of the targets are involved in a synergistic interaction and is slow to adapt to findings in the early phases of the screen. Concurrent estimates 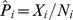 (no prior), on the other hand, are too sensitive to early findings in the screen. In principle, 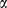 and 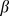 could be chosen in a gene-specific manner, but this is reserved for future work.

### Matrix completion for interaction scores

#### The interaction score matrix should be in the intersection of three sets

We view the interaction matrix 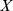 as an unknown point in the space of all real-valued symmetric matrices of size 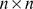, denoted Sym*_n_*. Our goal is to use different kinds of available evidence to define constrained subsets of Sym*_n_*, which contain the feasible values of 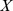. Given these subsets, we will predict 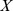 by finding a single feasible point that is located in the intersection of all three constrained subsets.

**The first subset**, 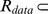
Sym*_n_*, contains all symmetric matrices that are consistent with our experimental observations. This set is defined by the sum of square distance from the experimental points, i.e.
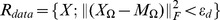
(8)where 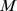 is the experimental data, and the notation 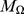 denotes that M is only determined (observed) for a subset 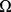 of matrix elements, which correspond to the known interaction scores. The norm 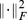 denotes the Frobenius norm (sum of the squared elements) for matrices, and 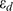 is an upper bound on the acceptable disagreement (tolerance) with the experimental data.

**The second subset**, 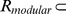
Sym*_n_*, is the subset of matrices that fulfill the criterion of having the characteristic ‘modular’ structure typically seen in interaction score data. Clusters of interaction scores are frequently attributed to shared biological functions. Previously, this principle has been used to interpret interaction scores as functional modules, or make predictions of the function of particular targets or drugs [2], [4], [24], [35]. Here, we instead aim to define a matrix algebraic constraint on 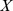 that ensures modularity. To define a set of symmetric matrices that have a modular structure, we apply the nuclear norm constraint
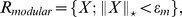
(9)where 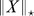 denotes the nuclear norm of 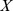, defined as the sum of the singular values of 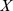. In practice, constraining the nuclear norm of a matrix is used as a technique to constrain the rank of 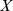 [22]. A small value of 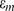 thus implies few modules in the data. Obvious alternatives to this constraint would be e.g. the monochromaticity score by [24] and likelihood-based scores [36] or constraining the rank of 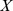. However, the nuclear norm, which is a convex function of 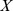, is very well suited for rapid optimization techniques [21], [22].

**The third subset**, 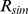, contains all matrices consistent with prior pathway information. In contrast to the previous subset, 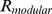, which contains *any* matrix with *any* modular structure, 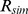 contains more specific information, i.e. it defines *a particular* modular structure defined by external data. We define this set by:
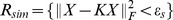
(10)Here, 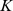 is a matrix that reflects the expected degree of similarity between rows and columns of 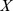. To motivate this definition, consider an unknown interaction score 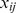 and a linear interpolation function that predicts 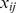 from any available “neighboring”, scores 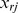, 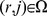. In other words,
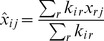
(11)here, 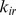 is a non-negative weight that quantifies the functional similarity between target 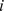 and 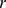. We organize these weights into a matrix format 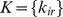 and scale the rows/columns to sum to 1, i.e. 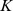 is a bistochastic matrix. We note that a fully observed 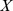 is consistent with the above kernel estimate if 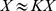, i.e. when 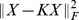 is small, which motivates the definition of the set 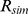 (eq. 10).

#### Functional similarity data

We explored which available data sources can be used to construct a matrix 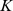 with the property that 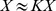. Here aiming for a heuristically defined 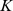, we first defined 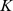 from different data sources, as the properly scaled matrix formed from (i) protein-protein interaction networks, (ii) co-expression networks, (iii) naïve GO term correlations; and, (iv) GO-term derived semantic scores [37]–[39] using 18 alternative tables from Yang et al. [40]. To gain insight about the usefulness of each data type as a prior to predict gene-gene interactions in yeast, we evaluated a total of 22 different 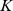-matrices (listed in Supplementary Table 2 in File S1) using the metric
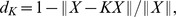
which will assume the value 0 if 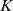 fails to capture the contents of 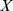 and 1 if 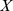 is perfectly explained by 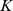. (We remind the reader that 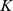 is a bistochastic matrix with zero diagonal, which excluded the trivial solution 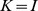, the identity matrix). Overall, the results show that PPI, GO term correlations and GO semantic scores were relatively equal in explanatory power (explaining up to 19% of 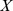, Supplementary Table 2 in File S1) and while mRNA from one particular compendium were slightly less efficient. In the simulations below, we thus computed the average 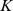 for PPI (MINT) [41], mRNA, GO correlation and GO semantic data. Averaging was done using identical weights for each of the data types. The possibility of readjusting such weights during an ongoing screen, is reserved for future work.

For the glioma experiments, we defined five functional groups among the 31 drugs (Supplementary Table 1 in File S1). We thus defined 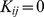 when drug were in two different groups, and 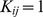 when they were in the same group. This matrix was subsequently scaled by bistochastic scaling.

#### Predicting interaction scores by cyclical projection onto convex sets

Our next task is to find an interaction matrix, which is located in the intersection of all three subsets in Sym*_n_*, i.e. it fits the data (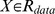), it is modular (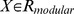); and, it is consistent with database information (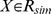). In other words,
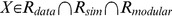
(12)There are highly efficient numerical methods to find the intersection of sets. Here, we find the solution by a cyclical sequence of projections, a method which has previously been applied to signal recovery and feasibility problems with multiple constraints [42]–[44].

Our algorithm starts with 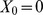 (a matrix with all entries equal to zero) and subsequently alternates between these three steps:
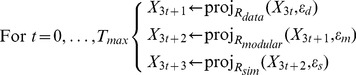
(13)where 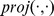 denotes projection onto (or towards) the respective set (in the Frobenius norm). Each function in equation 13 thus maps a point 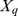 in the space of matrices to a new point 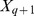, which lies within the current set of interest 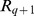 and also is at a minimal distance to the previous point 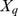.

We cycle over projections until a converge criterion is met (see below). If the sets have a nonempty intersection, convergence is guaranteed by that fact that the three operations are cyclically applied projections onto convex sets [42].

By default, our method starts from an initial guess of a matrix of zeroes. To assess the robustness of the algorithm to differences in the initial guess in matrix space (i.e. the starting point in figure 2A), we performed a simulation in which *Saccharomyces cerevisiae* data with 80% missing values were imputed, using randomized matrices as 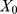 (each matrix containing iid normally distributed random values with 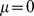 and 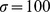). For each of the 100 simulations, the maximum deviation of 2 matrix elements between any two simulations was always less than 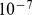, with a mean of 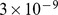, i.e. within the numerical precision (Figure S3 in File S1). This suggest that the performance of the algorithm is insensitive to the choice of initial condition.

Our method is a heuristic extension of previously described matrix completion algorithms Softimpute and SVT [21], [22], [26]. As a special case (relaxing constraints on modularity and functional similarity) our algorithm corresponds to the Softimpute algorithm [22]. The inclusion of functionality similarity is not a feature of this previous method, nor of the other methods considered in our comparison study (LLS [19] and EMDI [20]). Moreover, the general framework of cyclical projections we employ may have other extensions (e.g. by including additional convex set constraints for other data types), but this exploration is reserved for future work. All comparisons presented are based on Pearson correlation. However, using sum of squares prediction error did not alter the ranking of the tested methods.

### Implementation

Our cyclical projection algorithm, explained above, starts with an empty interaction score matrix 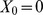 and subsequently applies three projection operations (onto the sets 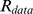, 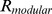 and 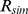) to obtain a sequence of iterates 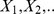 until convergence, here defined as a small fractional change of 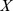 in terms of the Frobenius norm, i.e. 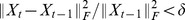, with 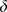 set to 0.0001.

For practical purposes, the projection functions (eq. 13) are not parameterized with the tolerance constants 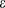 (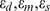) used to define the sets, but with penalties 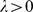. This has no consequence for the solution of the problem, since for a given 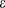 there is a 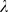 which produces the same solution and vice versa [45]. We provide the derivation of the explicit projection formulae and parametrization using 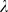 in the Supplement (Lemma 1–3 in File S1). The projection operations have the following computational forms:
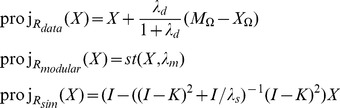
(14)


Here, 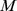 is the experimental data, and the notation 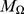 denotes that 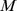 is only determined (observed) for a subset 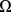 of matrix elements, which correspond to the known interaction scores. 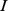 denotes the identity matrix and st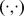 is a soft-thresholding operation on the singular values of 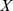, defined as 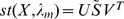, where 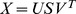 is the singular value decomposition (SVD) of 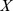, and 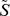 is defined as 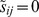 for 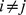, and 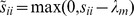 [21], [22].

We implemented this algorithm in MATLAB, using the PROPACK package to calculate the Singular Value Decompositions necessary for projection onto the set 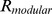. We choose 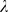 constants as follows. 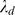 is kept constant at a default value of 10 (changing this value did not affect the results in a significant manner, although values close to zero should be avoided as 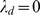 would imply that the experimental data are non-informative). 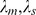 are chosen by five-fold cross-validation, in which 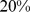 of data points in 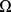 are left out and predicted for a series of (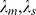) pairs. We chose the pair that maximizes predictive power, measured by the Pearson correlation between observed and predicted values.

One should also note that for some choices of 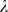, the three 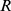 sets will become too small, and not overlap; in such cases the algorithm will instead converge onto a limit cycle, alternating between a limited number of solutions. In these cases, we recommend decreasing the 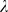 values, alternatively using the average 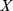 over the three cycling steps as a solution that lies close to all sets [42].

In terms of algorithmic speed, the most time-demanding step is the calculation of the first few components of a singular value decomposition (SVD) of 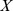 (to project onto 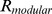). The projections onto 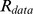 and 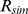 merely require matrix multiplications. The MATLAB implementation uses the PROPACK package to compute the SVD. As an example of a running time, the method requires 2 seconds to converge for a 500 matrix and about 10 minutes for a 4000 matrix. However, in cases where 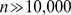 improved SVD methods are needed and we consider adding this to future versions of the implementation. The code is available from the authors upon request.

### Screening via propensity-based sampling and interaction score prediction, Algorithms 1 and 2

Algorithm 1 outlines the screening strategy that incorporates prior knowledge or observed marginal interaction propensity for each gene or drug, i.e. how frequently an individual target is involved in a synergistic interaction. Algorithm 1 consists of iteration of steps 1 and 2 below. Algorithm 2 is the screening principle that incorporates both marginal propensity and interaction score prediction via matrix completion. Algorithm 2 is defined via steps 1 through 5. Two tuning parameters, 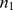 and 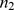, determine the number of experiments to perform in each step of the screen. While it is theoretically possible to step through the screen one experiment at a time, it is probably not the most practical strategy. As a default we used 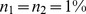 of all pairs.

Propensity-based sampling: steps 1 and 2.

1. Estimate probabilities 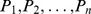 for the targets to have synergism with any other target (see main text for definition of Bayes estimates of 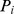).
2. Perform 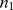 experiments sampled from the untested fraction experiments, where the sampling probability for each pair is proportional to 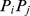.

Prediction-driven screening: steps 3 and 4.

3. Use the matrix completion method defined by equations (14) to predict interaction scores 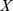.
4. Pick the 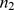 most extreme predicted interactions and test them by experiment.

Switching between the propensity-based and prediction-driven paradigms.

5. Estimate the hit rates 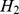 for the 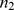 most recent prediction-based experiments and 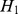 for the 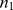 previous random experiments (sampled as in step 2). If 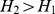 go to step 3. Otherwise go back to step 1. The hit rates are defined as the ratio between the number of identified synergies and the total number of experiments.

## Supporting Information

File S1 **Supporting figures and tables.**
(PDF)
