# Supplementary material for: Searching for Synergies: Matrix Algebraic Approaches for Efficient Pair Screening
Source: PLoS One. 2013 Jul 25;8(7):e68598. doi: 10.1371/journal.pone.0068598 (PMC3723843; doi:10.1371/journal.pone.0068598)
Supplement: File S1 — Supporting figures and tables. (PDF) [file pone.0068598.s001.pdf]

Supplementary information for "Searching for synergies: matrix algebraic approaches for efficient pair screening"

Derivation of projection operations

In this section we demonstrate that the projections on the three subsets R_{data}, $R_{modular}$ and R_{sim} are given by:

$$\begin{array}{l} \mathrm{proj}_{R_{data}}(X) = X + \frac{\lambda_1}{1+\lambda_1}(M_\Omega - X_\Omega) \\ \mathrm{proj}_{R_{modular}}(X) = \mathrm{st}(X, \lambda_2) \\ \mathrm{proj}_{R_{sim}}(X) = (I - ((I-K)^2 + I/\lambda_3)^{-1}(I-K)^2)X \end{array} \tag{1}$$

where $\mathrm{st}(\cdot)$ stands for soft-thresholding defined below.

Lemma 1: projection onto R_{data}

The set R_{data} is defined as

$$R_{data} = \{X; \|(X_\Omega - M_\Omega)\|_F^2 < \varepsilon\} \tag{2}$$

The projection of X (in the Frobenius norm) onto R_{data} is a point $X+\delta$ which satisfies

$$\arg\min_{\delta} \|\delta\|_F^2 \ \text{ such that } \ \|((X+\delta)_\Omega - M_\Omega)\|_F^2 < \varepsilon. \tag{3}$$

Instead consider the closely related problem

$$\arg\min_{\delta} \ \|\delta\|_F^2 + \lambda_1 \|((X+\delta)_\Omega - M_\Omega)\|_F^2 \tag{4}$$

The two problems are in fact equivalent, since for a given ε, it is possible to find a $\lambda_1 > 0$ so that the two problems share the same solution, and vice versa [1]. Differentiation with respect to δ, and setting the derivative to zero, yields:

$$\delta + \lambda_1((X+\delta)_\Omega - M_\Omega) = 0 \tag{5}$$

Solving for δ yields

$$\delta = \frac{1}{1+\lambda_1}(M-X)_\Omega \tag{6}$$

So the projected point becomes

$$\mathrm{proj}_{R_{data}}(X) = X + \delta = X + \frac{\lambda_1}{1+\lambda_1}(M_\Omega - X_\Omega) \tag{7}$$

Lemma 2: projection onto $R_{modular}$

The set $R_{modular}$ is defined as

$$R_{modular} = \{X; \|X\|_* < \varepsilon'\} \tag{8}$$

The projection of X (in the Frobenius norm) onto $R_{modular}$ is a point $X+\delta$ which satisfies

$$\arg\min_{\delta} \|\delta\|_F^2 \ \text{ such that } \ \|X+\delta\|_* < \varepsilon'. \tag{9}$$

Please note that although the nuclear norm is used for defining the convex target set, it is the Frobenius norm which is used for the projection. Again, we note that this problem is equivalent to a similar convex problem:

$$\arg\min_{\delta} \ \|\delta\|_F^2 + \lambda\|X+\delta\|_* \tag{10}$$

We apply the variable changes $Z = X + \delta$, whereby $\delta = Z - X$. Then we can express our optimization problem as

$$\min_{Z} \|Z - X\|_F^2 + \lambda \|Z\|_* \tag{11}$$

We can then apply the result by Mazumder et al. [2], whereby this problem is solved by

$$\hat{Z} = \text{st}(X, \lambda_2). \tag{12}$$

Here $\text{st}(\cdot,\cdot)$ is a soft-thresholding operation on the singular values of X, defined as $\text{st}(X, \lambda_2) = U\tilde{S}V^T$, where $X = USV^T$ is the singular value decomposition (SVD) of X, and $\tilde{S}$ is defined as $\tilde{s}_{ij} = 0$ for $i \neq j$, and $\tilde{s}_{ii} = \max(0, s_{ii} - \lambda_2)$. In conclusion, our projected point is

$$\text{proj}_{R_{modular}}(X) = X + \delta = Z = \text{st}(X) \tag{13}$$

Concerning the projection on $R_{modular}$, we get very similar results when we project onto a matrix with a specific rank k, by:

$$\text{proj}_{R_{modular}}(X) = U_k S_k U_k^T \tag{14}$$

where U_k is the $n \times k$ matrix containing the k first singular components and S_k is a diagonal matrix with the k largest singular values. This is the standard least squares fit to a k-dimensional subspace, calculated from symmetric singular value decomposition of X (i.e. $X = USU^T$, with $UU^T = I$ and S being diagonal).

Lemma 3: projection on R_{sim}

Again, we take the appoach of formulating the projection as a convex problem, which now becomes:

$$\arg\min_{\delta} \|\delta\|_F^2 + \lambda \|(X + \delta) - K(X + \delta)\|_F^2 \tag{15}$$

Rearranging this expression into:

$$\arg\min_{\delta} \|\frac{1}{\sqrt{\lambda}} I\delta\|_F^2 + \|(I - K)\delta - (-(I - K)X)\|_F^2 \tag{16}$$

The problem is equivalent to Tikhonov-regularized least squares solution for δ, with solution given by

$$\hat{\delta} = -((I - K)^T(I - K) + \tfrac{1}{\lambda} I)^{-1}(I - K)(I - K)^T X \tag{17}$$

Since K is symmetric, it simplifies to

$$\hat{\delta} = -((I - K)^2 + \tfrac{1}{\lambda} I)^{-1}(I - K)^2 X \tag{18}$$

So the projected point becomes

$$\begin{aligned} &\text{proj}_{R_{sim}}(X) = X + \delta = \\ &(I - ((I - K)^2 + I/\lambda)^{-1}(I - K)^2)X \end{aligned} \tag{19}$$

Functional similarity data

We computed a functional similarity matrix K (meant to represent a prior structure of modularity in the system) from different data sources. For PPI data, we obtained yeast PPI links from Intact and MINT via the pathwaycommons server (`pathwaycommons.org`), and defined a matrix W, where w_{ij} = 1 if there is a PPI, and 0 otherwise, and $w_{ii} = 0$ for all diagonal elements. This was followed by $K = f(W)$, where $f(\cdot)$ is a function that first sets all diagonal elements to zero, followed by scaling to bistochasticity. For mRNA data, we calculated the Pearson correlation r between all mRNA pairs i,j in the GEO data set, and defined $w_{ij} = 1 - r_{ij}$, followed by scaling $K = f(W)$. For naïve GO correlations, we used defined $w = HH^T$, where H is a matrix of genes times GO terms with $h_{rs} = 1$ when gene r has GO term s, followed by scaling $K = f(W)$. Finally, we considered pre-computed semantic similarity matrices SIM1,SIM2, etc. from the paper by Yang et al. [7], followed by scaling $K = f(W)$. The performance of the different similarity measures is shown in supplementary table 2.

Supplementary tables

Supplementary Table 1: The metric shown for each matrix is given by $d_K = 1 - \|X - KX)\|/\|X\|$, which will assume the value 0 if K fails to captures the contents of X and 1 if X is perfectly explained by K. Note that since K has zeros along the diagonal, the trivial case of $K =$ identity matrix is not possible.

Matrix K	Prediction metric, d_k	Source
PPI (Intact)	0.1372	[3]
PPI (MINT)	0.1426	[4]
mRNA (GEO)	0.0884	[5]
GO corr	0.1916	[6]
'SIM1'	0.1203	[7]
'SIM2'	0.1265	[7]
'SIM3'	0.1101	[7]
'LINSIM1'	0.1195	[7]
'LINSIM2'	0.1245	[7]
'LINSIM3'	0.1101	[7]
'JIANGSIM1'	0.1149	[7]
'JIANGSIM2'	0.1261	[7]
'JIANGSIM3'	0.0619	[7]
'RW18SIM1'	0.1213	[7]
'RW18SIM2'	0.1273	[7]
'RW18SIM3'	0.1102	[7]
'RW28SIM1'	0.1181	[7]
'RW28SIM2'	0.1281	[7]
'RW28SIM3'	0.0644	[7]
'RW38SIM1'	0.1209	[7]
'RW38SIM2'	0.1262	[7]
'RW38SIM3'	0.1103	[7]

Supplementary Table 2: Compounds used in the glioblastoma cell line experiments.The pathway groups are used to assess within-group and between group correlations of interaction scores, as analyzed in supplementary figure 1.

Compound	Target/mechanism	Pathway group	Supplier
Erlotinib	EGFR	RTK	DTP, Enzo
Gefitinib	EGFR	RTK	NCC, DTP, Selleck
Quercetin	PI-3K	PI3K/mTOR	BIOMOL
Wortmannin	PI-3K	PI3K/mTOR	BIOMOL
LY 294 002	PI-3K	PI3K/mTOR	BIOMOL, Enzo
ZM 336 372	cRAF	RAF	BIOMOL
GW 5074	cRAF	RAF	BIOMOL
Retinoic acid	RAR/RXR		BIOMOL
Doxorubicin	Intercalating cytostatic		DTP, Sigma
Imatinib	PDGFR, Bcr/Abl, c-KIT	RTK	NCC
Imipramine	SSRI	ANTIDEP.	NCC, Sigma
Sertraline	SSRI	ANTIDEP.	NCC
Temozolomide	Alkylating cytostatic		DTP, NCC
Olomoucine	Cycklin dependent kinases (CDK)	CDK	BIOMOL
Roscovitine	Cycklin dependent kinases (CDK)	CDK	BIOMOL, Enzo
Metformin	GLUT4		Enzo, Sigma
Fluperlapine	Antipsychotic		NCC, Enzo
Ipriflavone	Synthetic isoflavone		NCC, Sigma
Physostigmine	Cholinesterase		NCC, Enzo
Rapamycin	mTOR	PI3K/mTOR	BIOMOL, Enzo
Nelarabine	Purine nucleoside analogue/cytostatic		DTP, Tocris
Rimcazole	Sigma receptor		NCC, Tocris
Cefaclor	Cephalosporin antibiotic		NCC, Enzo
Pirenperone	5-HT2		NCC, Sigma
AG-490	JAK2		BIOMOL, Enzo
U-0126	MEK1 and MEK2		BIOMOL, Enzo
Paclitaxel	Cytostatic		DTP, Enzo
Zolmitriptan	Serotonin receptor agonist		NCC, Enzo
Piroxicam	NSAID		NCC, Enzo
Pterostilbene	Stilbenoid		NCC, Enzo
PP2	Src		BIOMOL, Enzo

NCC = National Clinical Collection from National Institute of Health (NIH), DTP = Developmental Therapeutic Program Oncology drug plate supplied by NIH, Enzo = Enzo Biosciences, Sigma = Sigma Aldrich, BIOMOL = BIOMOL plate from Enzo, Selleck = SelleckChem, Tocris = Tocris Biosciences

Supplementary figures

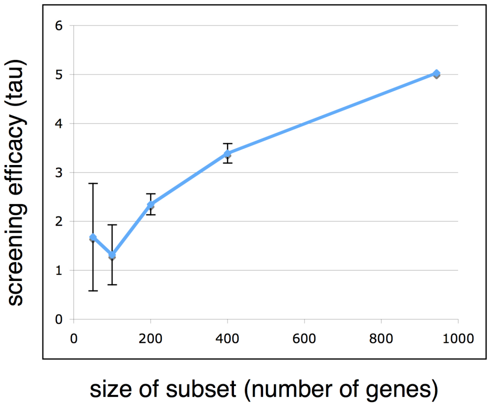

Figure S 1: **Screening efficacy τ for different subsets of the Constanzo et al (2010) data.** Theoretically, we expect screening efficacy to increase with the number of targets. To confirm this trend, we used random subsets of the Costanzo et al (2010) data of size 50, 100, 200 etc genes. Curve: average τ for 10 randomly drawn subsets. Error bars are standard deviation.

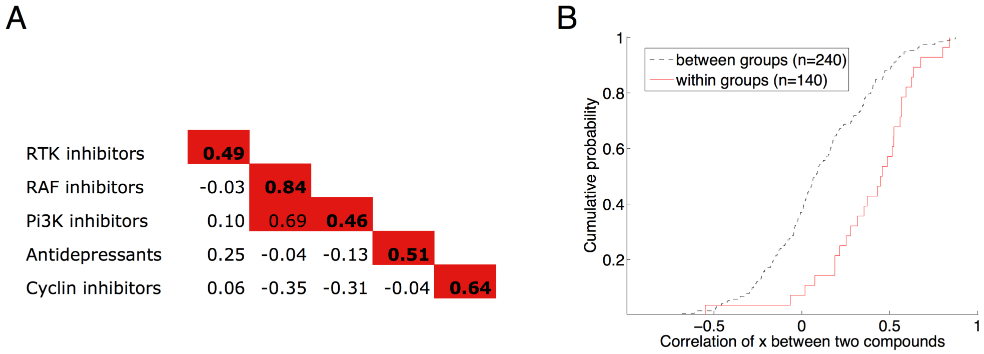

Figure S 2: **Assessment of functional modularity of drug-drug interactions in five glioblastoma cell lines.** From the 31 compounds used in our glioblastoma cell line experiments (c.f. Figure 3), we defined four subgroups of 2-4 compounds, listed in supplementary table 1. If interaction scores are functionally modular, as assumed in our model, the interaction scores should correlate more within groups (diagonal boxes) than between groups (white boxes). Red indicate a pearson correlation greater than 0.45, note that correlation is typically higher along the diagonal (within groups). B) the cumulative distribution of all pairwise comparisons is clearly shifted to the right within groups, (Kolmogorov-Smirnov test p-value less than 10^{-20}.

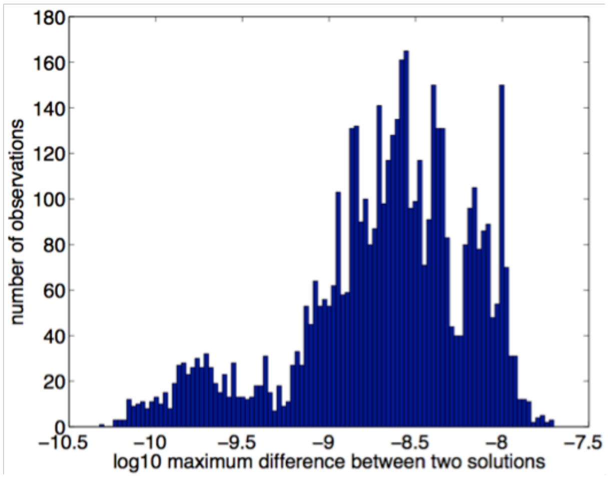

Figure S 3: **The performance of the projection algorithm under different initial conditions.** A histogram of the quantity $\|X_a - X_b\|_\infty$, i.e. the largest element-wise difference between any two pairs of realisations with random initial matrix X_0 (each matrix containing iid normally distributed random values with $\mu = 0$ and $\sigma = 100$).

References

[1] Osborne MR, Presnell B, Turlach BA (2000) On the lasso and its dual. J Comput Graph Statist 9: 319–337.

[2] Mazumder R, Hastie T, Tibshirani R (2010) Spectral regularization algorithms for learning large incomplete matrices. J Mach Learn Res 11: 2287–2322.

[3] Kerrien S, Aranda B, Breuza L, Bridge A, Broackes-Carter F, et al. (2012) The intact molecular interaction database in 2012. Nucleic Acids Res 40: D841–D846.

[4] Ceol A, Chatr Aryamontri A, Licata L, Peluso D, Briganti L, et al. (2010) Mint, the molecular interaction database: 2009 update. Nucleic Acids Res 38: D532–D539.

[5] Moser JJ, Fritzler MJ (2010) The microrna and messengerrna profile of the rna-induced silencing complex in human primary astrocyte and astrocytoma cells. PLoS One 5: e13445.

[6] Ashburner M, Ball CA, Blake JA, Botstein D, Butler H, et al. (2000) Gene ontology: tool for the unification of biology. the gene ontology consortium. Nat Genet 25: 25–29.

[7] Yang H, Nepusz T, Paccanaro A (2012) Improving go semantic similarity measures by exploring the ontology beneath the terms and modelling uncertainty. Bioinformatics 28: 1383–1389.
